# Glymphatic function and astrocyte reactivity potentiates tau load in Alzheimer's disease

**DOI:** 10.1002/alz70856_102832

**Published:** 2025-12-24

**Authors:** Min Chu, Liyong Wu, Pedro Rosa‐Neto

**Affiliations:** ^1^ Xuanwu Hospital, Capital Medical University, Beijing, China; ^2^ Douglas Mental Health University Institute, Montreal, QC, Canada; ^3^ McGill University Research Centre for Studies in Aging, Montreal, QC, Canada; ^4^ McGill University, Montreal, QC, Canada; ^5^ Montreal Neurological Institute, Montreal, QC, Canada

## Abstract

**Background:**

Alzheimer's Disease (AD) is characterized by pathological protein deposition, and recent research suggests that glymphatic dysfunction and inflammation play critical roles in its pathogenesis. However, their direct interaction and impact on protein aggregation in AD have not been directly tested.

**Methods:**

We enrolled a cohort including individuals with cognitive impairment (CI, including AD and mild cognitive impairment MCI) and cognitively normal controls. We evaluated glymphatic function using DTI‐ALPS and inflammation through plasma GFAP, sTREM2, and CSF neuroinflammation markers.

**Results:**

Participants with lower ALPS levels showed altered inflammation profiles, specifically higher plasma GFAP and different CSF inflammation markers. There was a significant negative correlation between ALPS and plasma GFAP, and a positive correlation between ALPS and certain CSF inflammation markers. Moreover, glymphatic function and astrocyte reactivity synergistically potentiated tau load.

**Conclusion:**

Our findings suggest a relationship between glymphatic dysfunction and inflammation in AD, with implications for therapeutic interventions targeting both processes.

